# International Attention for Zoonotic Infections

**DOI:** 10.3201/eid1212.060000

**Published:** 2006-12

**Authors:** Nina Marano, Paul Arguin, Marguerite Pappaioanou, Bruno Chomel, Esther Schelling, Vincent Martin, Jay C. Butler, C. Ben Beard, Lonnie King

**Affiliations:** * Centers for Disease Control and Prevention, Atlanta, Georgia, USA;; † University of Minnesota, Minneapolis, Minnesota, USA;; ‡ University of California, Davis, Davis, California, USA;; § Swiss Tropical Institute, Basel, Switzerland;; ¶ Food and Agricultural Organization of the United Nations, Rome, Italy

**Keywords:** zoonotic infections, emerging diseases, exotic pet trade, intersectoral research, wildlife reservoir, microbial threats to health, introduction

**Figure 1:**
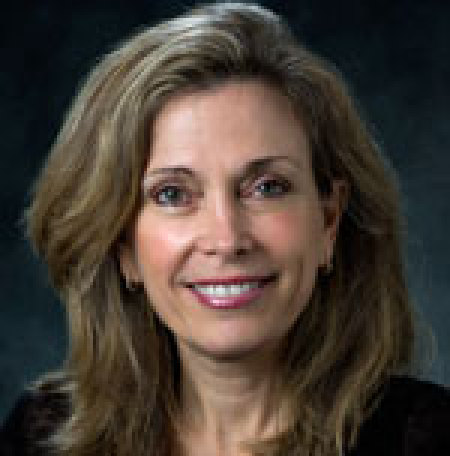
Guest editor Nina Marano. Dr Marano is the chief of the Geographic Medicine and Health Promotion Branch in the Division of Global Migration and Quarantine at CDC. The branch mission is to protect the health of US travelers at home and abroad and to prevent the introduction of zoonotic diseases into the country through imported animals and animal products.

**Figure 2:**
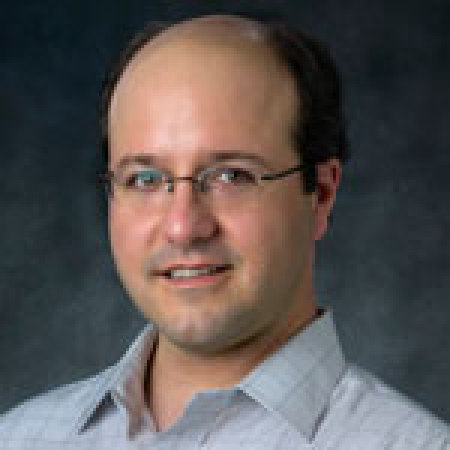
Guest editor Paul Arguin. Dr Arguin is the chief of the Domestic Response Unit in the Malaria Branch within the National Center for Zoonotic, Vectorborne, and Enteric Diseases at CDC.

**Figure 3:**
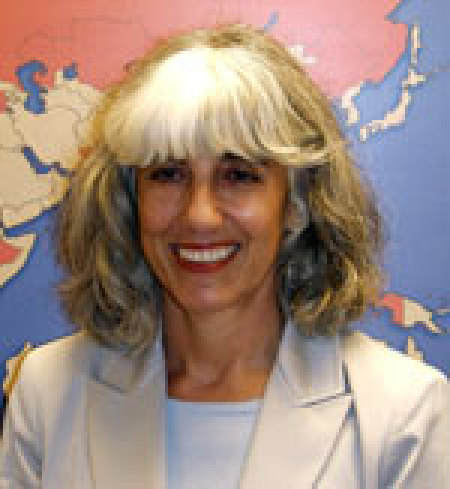
Guest editor Marguerite Pappaioanou. Dr Pappaioanou is professor of infectious disease epidemiology in the School of Public Health with a joint appointment in the College of Veterinary Medicine at the University of Minnesota. Her areas of interest are in emerging zoonotic infectious diseases, with a special interest in influenza viruses and in collaborative efforts that bridge public health and domestic animal and wildlife health sectors that address emerging zoonotic infectious diseases.

**Figure 4:**
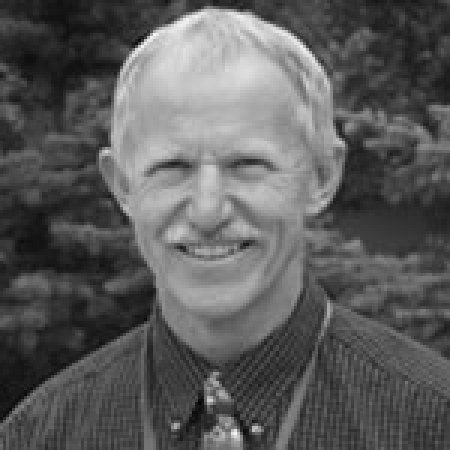
Guest editor Jay C. Butler. Dr Butler is on detail from CDC to the Alaska Division of Public Health, where he is deputy director for science and medicine and is the state epidemiologist. His research interests include vaccine-preventable diseases, antimicrobial drug resistance, emerging infectious diseases, microbial ecology, and emergency preparedness planning and response.

Guest Editors: Nina Marano (Figure 1), Paul Arguin (Figure 2), Marguerite Pappaioanou (Figure 3), and Jay C. Butler (Figure 4).

Snakes on a Plane was poised to be the blockbuster movie of the 2006 summer season. It featured reptiles smuggled onto an airplane as an unusual bioweapon of mass destruction. In a much less dramatic fashion, this absurd scenario plays out every day, when tons of live animals and unprocessed animal products are shipped internationally around the globe, providing many opportunities for rapidly translocating zoonotic pathogens. Episodes of emerging zoonoses are being increasingly recognized around the world. From 1996 to 2004, some 21% of 10,490 reports of animal diseases from 191 countries submitted to the Program for Monitoring Emerging Diseases (ProMED) concerned humans affected by zoonotic disease (*1*). This zoonoses theme issue of Emerging Infectious Diseases (EID) corroborates this finding, presenting reports of zoonotic disease from all corners of the globe, including the People's Republic of China, Vietnam, Slovakia, Indonesia, the United States, Israel, Bangladesh, the Netherlands, Brazil, Algeria, India, the Democratic Republic of the Congo, and Italy.

As public health and animal health organizations attempt to respond to these emerging and reemerging zoonotic diseases, their ability and skill in forming new strategic partnerships are of paramount importance. This year was highlighted by the establishment of the Centers for Disease Control and Prevention (CDC) as a World Organization for Animal Health (OIE) Collaborating Center for Emerging and Remerging Zoonoses.

To initiate this collaborating center, the International Symposium on Emerging Zoonoses (ISEZ) was held in conjunction with the International Conference on Emerging Infectious Diseases and the International Conference on Women and Infectious Diseases in Atlanta in March 2006. ISEZ was cosponsored by OIE, the United Nations Food and Agriculture Organization, the US Department of Agriculture Animal and Plant Health Inspection Service, the Department of Interior US Geological Survey, National Wildlife Health Center, the World Health Organization, and CDC. ISEZ was attended by >400 veterinarians, physicians, and public health professionals from all over the world. The objective of the symposium was to share information among public health and animal health professionals so that more effective and cooperative partnerships could be developed. In turn, this would help to better understand, prevent, and control new microbial dangers to human and animal health that occur globally every day.

The 44 internationally renowned ISEZ speakers and moderators recognized the spirit in which this meeting was created. Their participation reflected a true commitment to the partnership between public health and animal health needed to meet the ever-growing microbial challenges threatening human, animal, and environmental health on a seemingly continual basis. The speakers lent their talent and expertise to the following themes: the epidemiology of pathogens and diseases shared among humans and animals, the risks of wildlife and exotic pet trade to human and animal health, the effects of agricultural practices on human and ecosystem health, the lessons learned from previous experiences, and collaborative achievements. Speakers offered solutions that had been tested through innovative, interdisciplinary, and intersectoral research to deal with the problems of emerging zoonoses. A few presentations are highlighted below.

In his presentation, Emerging and Remerging Zoonoses from Wildlife Reservoirs to Exotic Pets, Bruno Chomel, University of California, Davis, College of Veterinary Medicine, reminded participants that most emerging infectious diseases are zoonotic, with wildlife constituting a large and often unknown reservoir. Wildlife can also be a source for reemergence of previously eradicated zoonoses. The discovery of such zoonoses is often related to better diagnostic tools. However, human modification to natural wildlife habitats and human behavior, such as deforestation, which causes range expansion of the tick vector for Kyasanur Forest disease in India, also create opportunities for emergence of zoonotic diseases. Translocation of raccoon dogs from Asia into Europe has provided a new potential rabies reservoir. Human behavior includes sleeping with pets, reported by 30% of Americans, and keeping of exotic pets that provide opportunities for microbial transmission that have not existed until recently. More than 10,000 pet tigers are kept in the United States—more tigers than are currently living in the wild throughout the world. Thus, addressing human influences on ecosystems and behavior with regard to wild and exotic animals must be incorporated into efforts at preventing and controlling emerging diseases that involve wildlife and other valued natural resources.

In her presentation, Combined Vaccination Delivery to Remote and Mobile Pastoral Families and Their Animals, Esther Schelling, Swiss Tropical Institute, focused on the potential of joining public health and veterinary services to achieve higher vaccination coverage in Africa's remote rural settings. Such sharing is aimed at reducing vaccine-preventable diseases in persons and their livestock. Her findings from Chad indicated that sharing transport logistics and cold chain equipment between the public health and veterinary sectors in the most remote areas reduced total program delivery costs, was highly valued by pastoralists, and was an important strategy to connect hard-to-reach populations with needed vaccinations such as for measles and polio for children and anthrax for livestock. By optimizing the use of limited logistic and human resources, public health and veterinary services were strengthened, especially at the district level, and, in turn, the services can become more prepared to respond to endemic and epidemic diseases.

ISEZ participants learned about the impact of human behavior on emergence and subsequent spread of highly pathogenic avian influenza (HPAI) H5N1 strain in Asia from Vincent Martin of the Food and Agriculture Organization. Human behavior that supported disease emergence includes agriculture practices that allow species mixing, limited biosecurity to prevent mixing of domestic and wild animals, and a cultural preference for warm meat that has made live bird markets common in urban areas. These factors act synergistically with the intrinsic characteristics of the virus and the rapid evolution of animal and farming production systems in the region to support emergence. Martin emphasized that understanding the underlying farming practices and cultural preferences that influenced the emergence and spread of HPAI in developing countries has been instrumental in implementing effective risk reduction measures. The experience of fighting HPAI in Asia shows that control efforts should be focused on production sectors with low biosecurity standards and free-ranging chickens and ducks. Creating conditions that are not conducive to microbial selection and emergence represents a major challenge, from a disease management and cultural point of view, for reducing the risk for avian influenza occurrence and subsequent human infection.

This issue of EID continues in the tradition of the 2 previous December theme issues by focusing on zoonoses, reminding readers again that we must maintain a vigilance in combatting these microbial threats. The issue highlights efforts directed at identifying disease reservoirs and finding better ways to understand, evaluate, prevent, and control disease transmission. At the same time, healthy ecosystems need to be promoted, economic livelihoods safeguarded, and cultural beliefs respected at household to national and international levels. Contained in this issue are articles based on the following ISEZ presentations: Review of Bats and SARS (*2*) and Ecologic Niche Modeling and Spatial Patterns of Disease Transmission (*3*). Also featured are articles that focus on the role of risk factors for introducing zoonotic diseases, such as monkeypox associated with domestic trade in certain animal species and human behavior as a risk factor for exposure to avian influenza in Vietnam. Many species of animals are highlighted, including cats colonized with methicillin-resistant Staphylococcus aureus, horses and wild mammals infected with West Nile virus, dogs with rickettsial infections, turkeys with human metapneumovirus, and nonhuman primates infected with malaria. We encourage our readers to continue to conduct and submit the findings of essential research on emerging zoonotic diseases to EID as we strive to share and disseminate this information to our multidisciplinary readership.
